# Immune function in paediatric trauma patients: a prospective explorative cohort study

**DOI:** 10.3389/fimmu.2026.1814648

**Published:** 2026-05-20

**Authors:** Lotte M. C. Jacobs, Michiel Vaneker, Demi van Dalen, Philip P. Horsting, Geert-Jan van Geffen, Freek Coumou, Manouk Backes, Lucas T. J. E. van Eijk, Leo A. B. Joosten, Michiel C. Warlé, Stijn D. Nelen

**Affiliations:** 1 Department of Surgery, Radboud University Medical Center, Nijmegen, Netherlands; 2 Department of Anaesthesiology, Radboud University Medical Center, Nijmegen, Netherlands; 3 Helicopter Emergency Medical Service, Radboud University Medical Center, Nijmegen, Netherlands; 4 Department of Orthopaedics, Sint Maartenskliniek, Nijmegen, Netherlands; 5 Department of Internal Medicine, Radboud University Medical Center, Nijmegen, Netherlands; 6 Department of Translational Immunology, Medfuture Institute for Biomedical Research, Iuliu Hatieganu University of Medicine and Pharmacy, Cluj-Napoca, Romania

**Keywords:** immunosuppression, inflammation, innate immune function, monotrauma, paediatric, polytrauma

## Abstract

**Background:**

Trauma is an important risk factor for the development of nosocomial infections. Immunological consequences of trauma in paediatric patients remains scarcely explored and associations between injury severity and immunosuppression, a decreased functionality of the immune system, have not yet been investigated in this population. Therefore, the aim of this study was to characterise the early effects of trauma and trauma severity on paediatric immune function, and to assess whether immune profiles differed between polytrauma patients who developed nosocomial infections and those who experienced an uncomplicated recovery.

**Methods:**

This prospective explorative cohort study was conducted at Radboud University Medical Center between January 2024 and June 2025. Three groups were included: controls (n=10), monotrauma patients (single fracture requiring acute surgery, n=9), and polytrauma patients (Injury Severity Score ≥ 16, n=10) aged 4–16 years. Immune function was assessed using blood samples at three timepoints: at the trauma scene (HEMS), at the emergency room (ER), and on post-injury day 1 (PID1). Immune outcomes included immune cell counts and functionality, plasma concentrations of damage-associated molecular patterns (DAMPs) and cytokines, and *ex vivo* cytokine production capacity upon whole blood stimulation with an endotoxin.

**Results:**

Inflammatory biomarkers were elevated already at the trauma scene, followed by compensatory mechanisms. Immunosuppression was already detected in the ER. Immune trajectories differed between poly- and monotrauma patients, with the latter showing a milder response. Polytrauma patients who developed nosocomial infections exhibited more profound immunosuppression. Immunosuppression was at least partially reversible *ex vivo* by co-stimulation with interferon-γ (IFN-γ).

**Conclusions:**

Paediatric traumatic injury rapidly elicits a robust immune response, particularly in cases of polytrauma, alongside compensatory mechanisms. Children who developed nosocomial infections showed more pronounced immunosuppression, which might be partially reversed with IFN-γ. Given the small sample size, these exploratory findings should be interpreted cautiously. Early immune monitoring may help identify paediatric trauma patients at increased infection risk who might benefit from immunomodulation.

**Trial registration:**

Medical Ethics Review Committee ‘METC Oost-Nederland’, file number 2023-16883.

## 1 Introduction

Every six minutes, a child arrives at an emergency department in the Netherlands due to an accident (1). While most traumatic injuries are minor, approximately 300 severely injured paediatric patients (Injury Severity Score (ISS) ≥ 16) are admitted to Dutch hospitals annually, and more than two-thirds of these children sustain severe traumatic brain injury (TBI) (2). Paediatric trauma is an important risk factor for the development of nosocomial infections, with the highest incidence observed in children with polytrauma and concomitant TBI (3, 4).

Traumatic injury induces an immune response. This response is elicited via the release of damage-associated molecular patterns (DAMPs). Binding of DAMPs to immune cells initiates the process of recruitment of monocytes and neutrophils aimed at promoting tissue repair (5, 6). This involves the release of pro-inflammatory cytokines (7) combined with a compensatory anti-inflammatory reaction (8, 9). The concurrent activation of pro- and anti-inflammatory responses illustrates the mechanisms that maintain immune homeostasis. A stronger initial inflammatory response is typically accompanied by a more pronounced compensatory reaction (10).

In adult patients, development of infections after traumatic injury has been linked to immunosuppression, a decreased functionality of the immune system (8, 11–15). Our research group found that severely injured adult patients already display immunosuppression at the trauma scene, just minutes to hours after injury (8). Immunosuppression is characterized by unresponsiveness of the immune system to pathogens and is associated with the development of post-traumatic nosocomial infections (8, 11–16).

In contrast to the adult population, the immune response and immunological consequences of trauma remain only scarcely explored in the paediatric population. Therefore, the aim of this explorative study was to characterise the effects of trauma and trauma severity on paediatric immune function, and to assess whether immune function differed between polytrauma patients who developed nosocomial infections and those who experienced an uncomplicated recovery.

## 2 Methods

### 2.1 Study population

This explorative prospective cohort study included paediatric patients with mono- or polytrauma and a control group. Exclusion criteria were age below 4 or above 16 years old, inability of blood sample collection due to clinical risks, known conditions that influence the immune response (e.g. auto-immune diseases or HIV), and use of medication that influences the immune response (e.g. high dose of steroids).

For the polytrauma group, inclusion criteria were an Injury Severity Score (ISS) ≥ 16 and involvement of the helicopter emergency medical service (HEMS, Lifeliner 3) at the trauma scene. The mono-trauma group consisted of children admitted with a single fracture requiring acute surgery. The control group comprised children who underwent elective posterior instrumented spinal fusion for idiopathic scoliosis.

The study was conducted in the Netherlands in accordance with the applicable regulations as determined by the recognized Medical Ethics Review Committee ‘METC Oost-Nederland’ (file number 2023-16883). Written informed consent was obtained from paediatric patients and/or their legal representatives.

### 2.2 Sample and data collection

**Figure 1:**
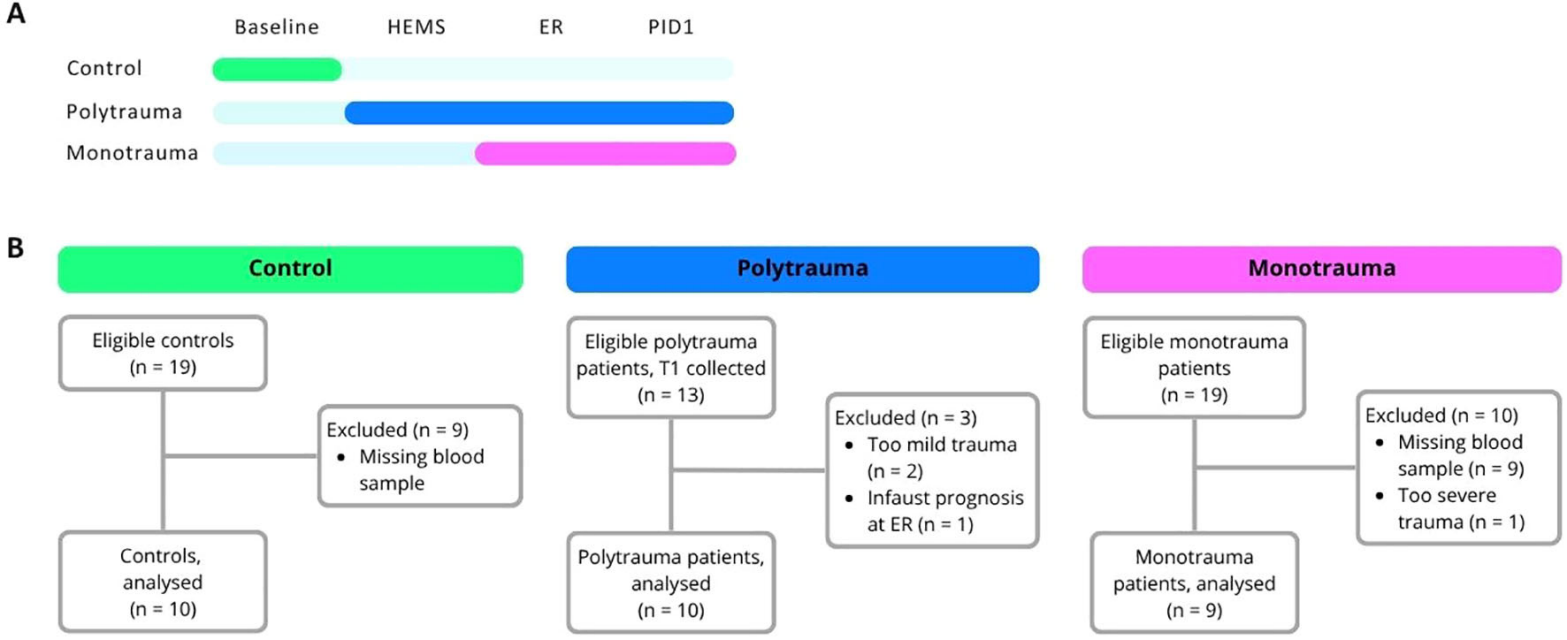
**(A, B)** Overview of blood sample timepoints **(A)** and participant flow diagram **(B)**. HEMS, helicopter emergency medical service; ER, emergency room; PID1, post-injury day 1.

Clinical data were obtained from digital patient files. Immune function was assessed using blood samples that were collected in tubes containing lithium heparin (LH) or ethylenediaminetetraacetic acid (EDTA) as anticoagulants at multiple timepoints (**Figure 1A**). In the polytrauma group, samples were obtained at the trauma scene shortly after the trauma by the HEMS, at the emergency room (ER), and on post-injury day 1 (PID1). The collection of blood samples did not impede, delay, or otherwise affect routine clinical care. Since the HEMS is not involved in mono-trauma cases, sampling in this group took place only in the ER and on PID1. In the control group, a blood sample was obtained prior to induction of anaesthesia (baseline). Blood cell counts were measured using a Sysmex haematology analyser.

All blood tubes were centrifuged at 2,970 Relative Centrifugal Force (RCF) for 10 minutes at room temperature. EDTA anti-coagulated plasma samples were centrifuged again at 16,000 RCF for 10 minutes at room temperature. Plasma was then stored at -80 °C until further analysis.

### 2.3 Plasma DAMP and cytokine concentrations

Plasma concentrations of the DAMPs heat-shock protein 70 (HSP70), S100A8/A9, and high mobility group box protein 1 (HMGB1) were measured using Enzyme-Linked ImmunoSorbent Assays (ELISAs) as specified in **Supplementary Table 1**. Plasma concentrations of the pro-inflammatory cytokines interleukin (IL)-6 and tumour necrosis factor (TNF), as well as the anti-inflammatory cytokine IL-10, were quantified using a Luminex assay according to the manufacturer’s instructions.

### 2.4 *Ex vivo* cytokine production capacity

Immune system functionality was assessed by measuring *ex vivo* cytokine production capacity after whole blood stimulation with Escherichia coli (E. coli) lipopolysaccharide (LPS) and/or interferon (IFN)-γ. Both stimulants were diluted in culture medium (RPMI 1640, Dutch modified) and added to LH-anticoagulated whole blood in 48-well flat-bottom plates at final concentrations of 10 ng/mL. Culture medium was supplemented with 50 µg/mL gentamicin, 2 mM Glutamax, and 1 mM pyruvate. After 24 hours of incubation at 37 °C, supernatants were collected and stored at -80 °C until further analysis.

Concentrations of pro-inflammatory cytokines IL-6, IL-1β, TNF, and anti-inflammatory cytokine IL-10 in supernatants were measured batch-wise using ELISAs according to the manufacturer’s instructions.

### 2.5 mHLA-DR expression

Human leukocyte antigen-DR expression on monocytes (mHLA-DR) was measured as a marker of monocyte activity and as an indicator of immunosuppression. EDTA-anticoagulated whole blood was incubated with QuantiBrite HLA-DR/Monocyte reagent. Following staining, red blood cells were lysed using FACS Lysing Solution. Samples were then acquired on a CytoFLEX flow cytometer.

Data were analysed using Kaluza Analysis software (version 2.3.0). Monocytes were identified by CD14 expression, and mHLA-DR expression was quantified using a mono-parametric histogram. The geometric mean fluorescence intensity of the total monocyte population was converted to the number of antibodies per cell (AB/C) using BD QuantiBriteTM PE Beads using the manufacturer’s instructions.

### 2.6 Statistical analysis

Continuous data in tables are presented as mean (range), and continuous data in figures as median (interquartile range, IQR). Categorical data are presented as numbers with percentages. Differences in plasma biomarkers and *ex vivo* cytokine production between the poly- and monotrauma groups, as well as comparisons between trauma patients and controls and between patients who developed nosocomial infections and those with a normal recovery were assessed using Mann–Whitney U tests. Due to the exploratory nature of this study, correction for multiple testing was not applied.

Statistical analyses were performed using SPSS statistics version 29 (IBM Corporation, Armonk, NY, USA) and RStudio version 2024.12.0 (Posit, PBC, Boston, MA, USA). Figures were created using Graphpad Prism version 10 (Graphpad Software, NY, USA) and RStudio. P-values < 0.05 were considered statistically significant.

## 3 Results

### 3.1 Patient characteristics

A total of 29 participants were enrolled between January 1^st^ 2024 and June 30^th^ 2025 (**Figure 1B**). Patients in the monotrauma group were younger than those in the polytrauma and control groups (**Table 1**). The polytrauma group consisted predominantly of males (80%), whereas the monotrauma and control groups showed a more balanced sex distribution (44% and 50% males, respectively). Most polytrauma patients sustained injuries to the head/neck or thorax.

**Table 1:** Patient characteristics.

Patient characteristics	Polytrauma (n = 10)	Monotrauma (n = 9)	Control (n = 10)
Age (years)	14 (12 - 16)	9 (4 - 16)	15 (14 - 16)
Male sex	8 (80%)	4 (44%)	5 (50%)
Length (m)	1.70 (1.52 - 1.93)	1.52 (1.21 – 1.69)	1.75 (1.62 – 1.91)
Weight (kg)	63.0 (32.0 – 90.0)	33.1 (16.0 – 56.2)	58.0 (50.4 – 85.0)
BMI (kg/m^2^)	19.4 (13.9 – 26.1)	15.6 (14.2 – 20.3)	19.4 (16.7 – 23.9)
American Society of Anesthesiologists (ASA) score	1 (1 – 1)	1 (1 – 2)	1 (1 – 2)
Injury Severity Score (ISS)	22 (17 - 33)	4 (4 – 9)	N/A
**Abbreviated Injury Score (AIS)** Head injury Neck injury Thorax injury Lower extremity injury Upper extremity injury	***AIS ≥ 3, n (%)*** 6 (60%) 3 (30%) 6 (60%) 1 (10%) 0 (0%)	***AIS ≥ 1 , n (%)*** 0 (0%) 0 (0%) 0 (0%) 3 (33%) 6 (67%)	N/A N/A N/A
PICU admission Mechanical ventilation^#^ Vasopressor therapy PICU stay (days)	9 (90%) 6 (60%) (70%) 4.5 (0 – 49)	0 (0%) N/A N/A N/A	N/A N/A N/A N/A
Receipt of blood products before collection of the final blood sample	1 (10%)	N/A	N/A
Hospital stay total (days)	11.5 (2 – 49)	1 (0 – 3)	N/A
Re-admission, < 30 days	1 (10%)	0 (0%)	N/A
**Nosocomial infection, < 30 days** Total Lower respiratory infection (LRI) Central line-associated bloodstream infection	4 (40%) 2 (20%) 2 (20%)	0 (0%) 0 (0%) 0 (0%)	N/A N/A N/A

Data are presented as median (range) or number (%). Vasopressor therapy always consisted of noradrenaline. ^#^In patients requiring mechanical ventilation, the first blood sample was drawn after the procedure.

### 3.2 Monocyte count and functionality

**Figure 2:**
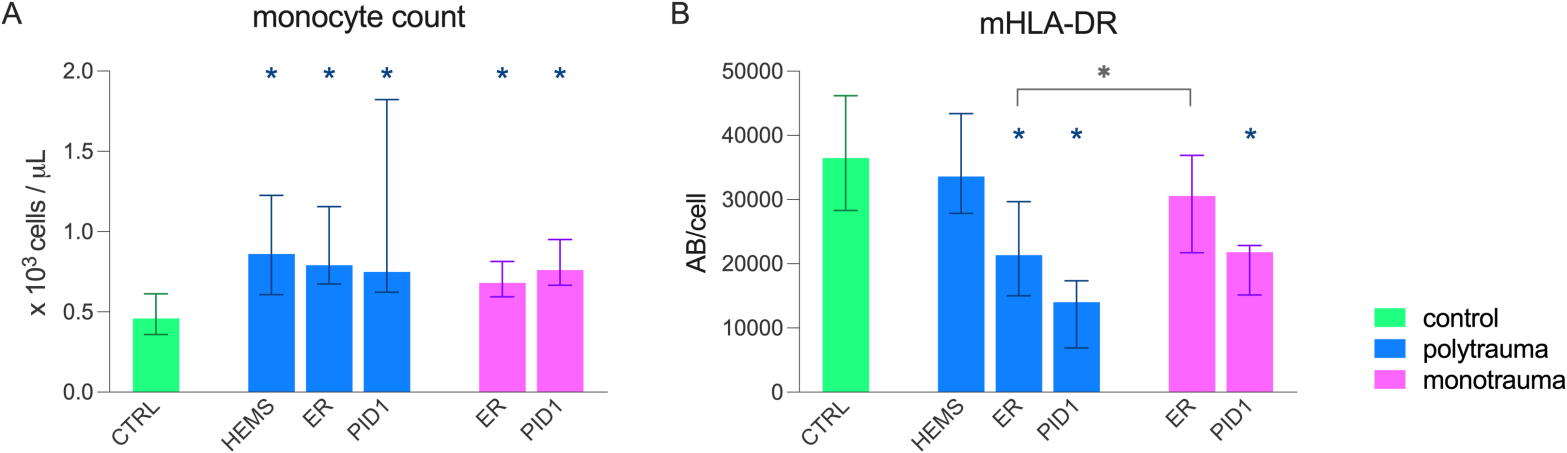
Monocyte count and monocytic Human Leukocyte Antigen – DR (mHLA-DR) expression in paediatric polytrauma (n = 10) and monotrauma (n = 9) patients at three timepoints (HEMS, helicopter emergency medical service; ER, emergency room; PID1, post-injury day 1) compared to controls (n = 10). **(A)** monocyte count, **(B)** mHLA-DR expression. Data are presented as median + interquartile range (IQR). * = statistically significant difference compared to control (p < 0.05); 
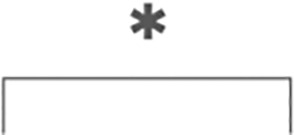
 = statistically significant difference between poly- and monotrauma groups (p < 0.05).

Monocyte counts were elevated at all timepoints in both poly- and monotrauma patients (**Figure 2A**). Expression of mHLA-DR was decreased compared with controls in the ER and PID1 in the polytrauma group (**Figure 2B**). In the monotrauma group, this decrease was observed only on PID1. mHLA-DR expression was lower in the polytrauma group compared to the monotrauma group in the ER (median [IQR]: 21,350 [15,039 – 29,619] vs. 30,545 [21,735 – 36,892] AB/cell, p = 0.035) but not on PID1 (median [IQR]: 14,032 [6,896 – 17,347] vs. 21,827 [15,130 – 22,872] AB/cell, p = 0.053).

### 3.3 Biomarkers in plasma

**Figure 3:**
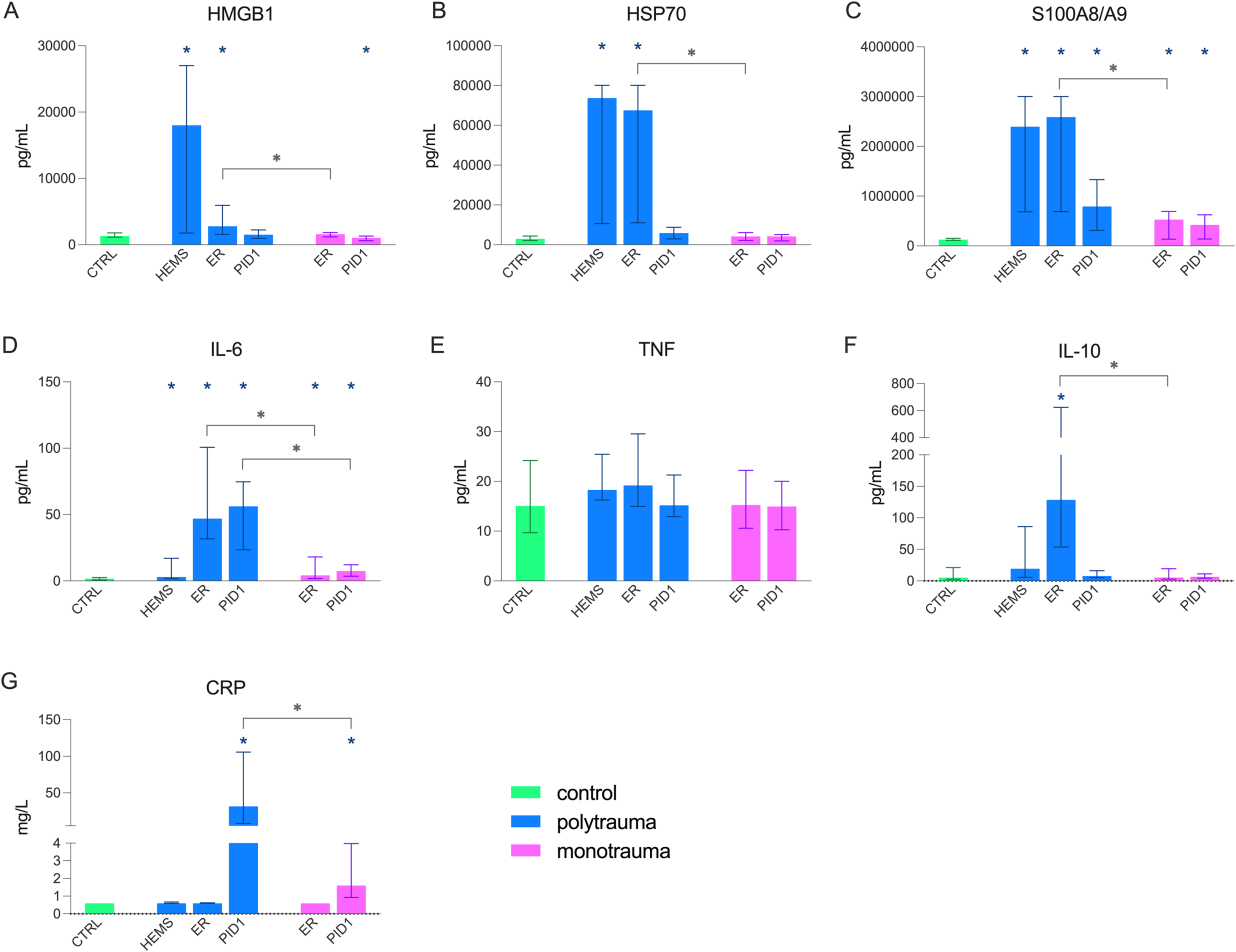
Concentrations of plasma biomarkers in paediatric polytrauma (n = 10) and monotrauma (n = 9) patients at three timepoints (HEMS, helicopter emergency medical service; ER, emergency room; PID1, post-injury day 1) compared to controls (n = 10). **(A)** High Mobility Group Box 1 (HMGB1), **(B)** Heat Shock Protein 70 (HSP70), **(C)** S100A8/A9, **(D)** interleukin (IL)-6, **(E)** tumour necrosis factor alpha (TNF), **(F)** IL-10, **(G)** C-reactive protein (CRP). Data are presented as median + interquartile range (IQR). * = statistically significant difference compared to control (p < 0.05); 
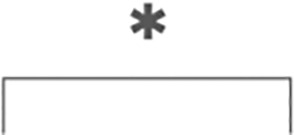
 = statistically significant difference between poly- and monotrauma groups (p < 0.05).

Plasma concentrations of DAMPs were elevated in trauma patients compared to controls (**Figures 3A–C**), with higher concentrations of HMGB1, HSP70, and S100A8/A9 in the polytrauma group compared to the monotrauma group in the ER. Concentrations of pro-inflammatory IL-6 were elevated in both trauma groups at all timepoints with higher concentrations in polytrauma patients versus monotrauma patients (**Figure 3D**), while TNF remained unchanged (**Figure 3E**). IL-10 was only elevated in the ER in polytrauma patients (**Figure 3F**). C-reactive protein concentrations were increased on PID1 in both groups, with significantly higher concentrations in polytrauma patients compared with the monotrauma group (median [IQR]: 31.6 [8.2 – 105.8] vs. 1.6 [0.9 – 4.0] mg/L, p = 0.003, **Figure 3G**).

### 3.4 Comparison of polytrauma patients with nosocomial infections and those with normal recovery

Four polytrauma patients developed nosocomial infections (**Table 1**): two developed lower respiratory infections and two developed central line-associated bloodstream infections, all occurring between PID3 and PID8. No deaths occurred during hospital admission or within 30 days following trauma. Subgroup analysis of patients with and without nosocomial infections revealed distinct differences between the two groups (**Supplementary Table 1**). Patients who developed infections had higher monocyte counts on PID1 compared with patients who experienced a normal recovery. mHLA-DR expression was consistently lower in the infection group across all three timepoints.

*Ex vivo* production of TNF, IL-6, and IL-10 following whole blood stimulation with LPS was more attenuated at the ER timepoint in patients who developed nosocomial infections. Additionally, on PID1, production of IL-1β and TNF was also more attenuated in patients who developed infections.

### 3.5 Reversal of immunosuppression using interferon-γ

**Figure 4:**
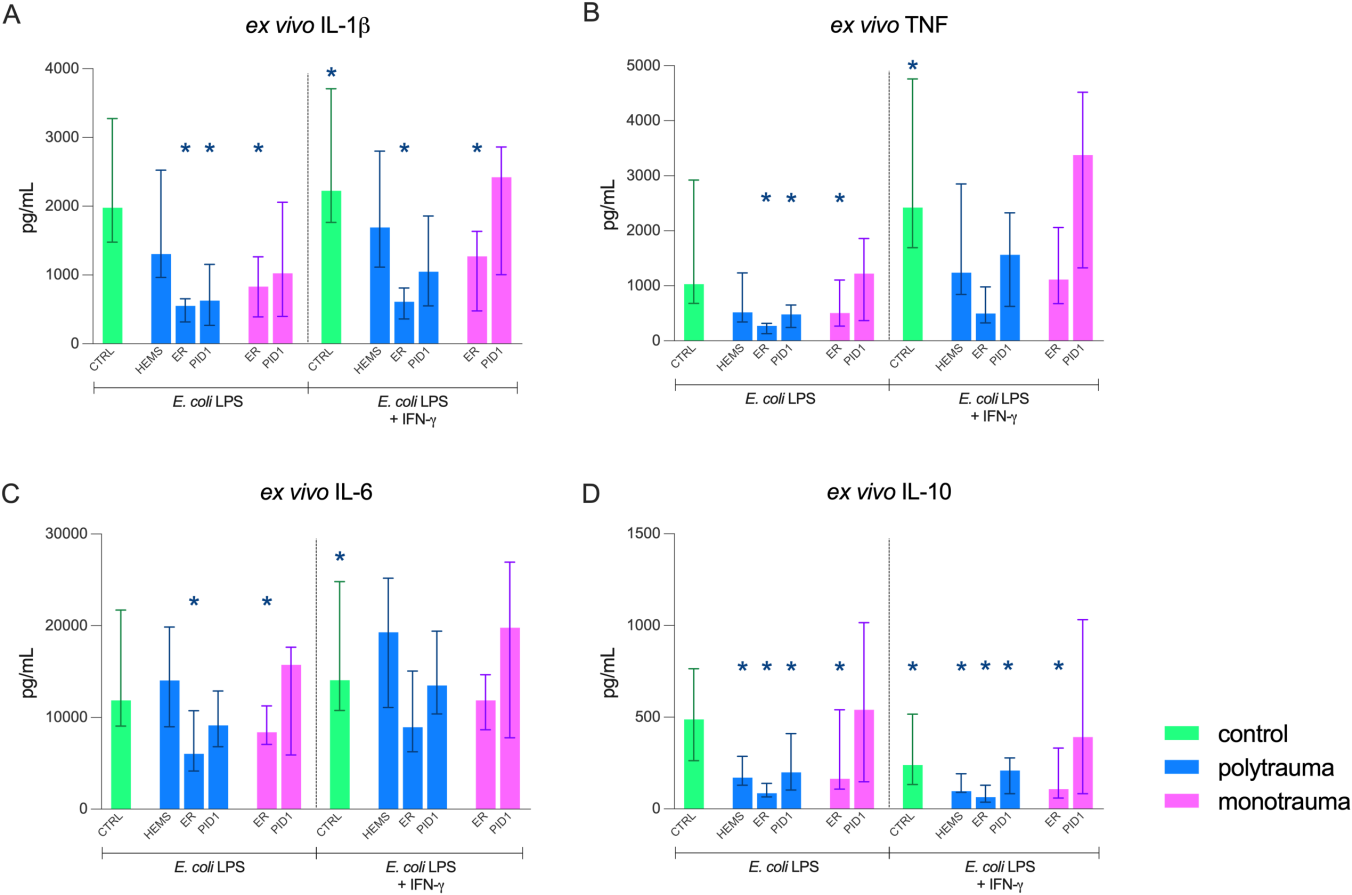
*Ex vivo* cytokine production of **(A)** interleukin (IL)-1β, **(B)** tumour necrosis factor (TNF), **(C)** IL-6), and **(D)** IL-10 upon whole blood stimulation with Escherichia coli (*E. coli* lipopolysaccharides (LPS) and interferon-γ (IFN-γ) in paediatric polytrauma (n = 10) and monotrauma (n = 9) patients at three timepoints (HEMS, helicopter emergency medical service; ER, emergency room; PID1, post-injury day 1) compared to controls without IFN-γ (n = 10). Data are presented as median + interquartile range (IQR). * = statistically significant difference compared to control (p < 0.05).

*Ex vivo* LPS stimulation revealed a marked attenuation of production of all pro-inflammatory cytokines (IL-1β, TNF, and IL-6) at the ER timepoint in the polytrauma group (**Figure 4**). In these patients, IL-1β and TNF production remained significantly suppressed on PID1, whereas IL-6 production had returned to normal levels. In the monotrauma group, production of all pro-inflammatory cytokines was reduced only in the ER, with full recovery by PID1. Anti-inflammatory IL-10 production was decreased at all timepoints in the polytrauma group, while in the monotrauma group IL-10 attenuation was limited to the ER timepoint.

IFN-γ co-stimulation significantly altered *ex vivo* cytokine production in nearly all samples. Exceptions were IL-10 at the ER and PID1 timepoints in the polytrauma group, IL-10 at PID1 in the monotrauma group, and IL-1β at the ER timepoint in the polytrauma group. TNF and IL-6 production in both trauma groups were restored to levels comparable to controls, but IL-1β production remained attenuated at the ER timepoint in both poly- and monotrauma patients. Production of IL-10 remained attenuated despite co-stimulation with IFN-γ.

## 4 Discussion

This study investigated the immune response to traumatic injury in paediatric patients, with additional focus on differences related to trauma severity and nosocomial infections. We found that trauma elicited a pronounced inflammatory response already at the trauma scene, followed by compensatory mechanisms. Immunosuppression was already detectable upon arrival at the emergency department, and appeared at least partially reversible by IFN-γ under controlled experimental conditions. Immune trajectories differed between poly- and monotrauma patients, with the latter showing a milder response. Additionally, polytrauma patients who developed nosocomial infections were observed to have more profound immunosuppression than those who experienced a normal recovery.

This study provides an in-depth characterisation of early immune dynamics following traumatic injury and is the first to include a pre-hospital timepoint in paediatric patients. While monocyte count increased following injury, monocyte function was reduced. Furthermore, we showed that predominantly polytrauma patients exhibited substantially elevated concentrations of DAMPs at the trauma scene and in the ER with subsequent elevations of plasma IL-6, IL-10, and CRP. The observed differences between patients and controls and effects of trauma severity were in line with previous studies (17–21).

Subgroup analysis suggested more severe immunosuppression in patients who developed nosocomial infections compared with patients who experienced a normal recovery, characterised by lower mHLA-DR expression and *ex vivo* cytokine production. Muszynski et al. also described lower TNF production in paediatric polytrauma patients (including burns) who developed nosocomial infections (22). On the contrary, mHLA-DR did not differ between patients with or without nosocomial infections on day 1 and day 3 in another paediatric polytrauma cohort (23). In that study, mean variations between day 1 and day 3, day 3 and day 8, and day 1 and day 8 also did not differ between the groups. However, in adult patients, Timmermans et al. described that a decrease in HLA-DRA expression between ER and day 3 was associated with the development of infections (8). Previous studies in adults have suggested 8,000 AB/cell as a threshold for severe immunosuppression (24, 25), In polytrauma patients who developed infectious complications, median mHLA-DR expression was below this limit. For *ex vivo* TNF production, thresholds vary depending on assay characteristics (26) and batch-variation. More research is needed to establish clinically relevant thresholds and to assess whether the observed immunosuppression in our study is reproducible in larger cohorts.

Our data provided evidence of immunosuppression in the ER, reflected by attenuated *ex vivo* cytokine production, which could (in part) be restored by concurrent stimulation with IFN-γ. To our knowledge, no previous studies have investigated the immunostimulatory effects of IFN-γ in paediatric trauma patients. Lendemans et al. showed that *in vitro* incubation of blood samples from adult polytrauma patients (ISS > 25) with IFN-γ enhanced mHLA-DR expression and *ex vivo* TNF synthesis (27). Furthermore, the use of immunostimulatory agents has mainly been studied in sepsis patients. In this patient group, IFN-γ administration reduced IL-6 and IL-10 levels and restored monocyte function (28). Murine models showed that IFN-γ reversed sepsis-induced immunosuppression via the PI3K/Akt/mTOR pathway (29). Taken together, these findings suggest that administration of IFN-γ might help reverse immunosuppression. However, larger and more comprehensive studies are required to further investigate the immunostimulatory effects of IFN-γ, as we did not assess other immune outcomes such as mHLA-DR expression after *ex vivo* co-stimulation. Moreover, additional research is needed to identify which patients are most likely to benefit clinically from immune modulation and to assess safety of immune modulation in these patients.

This study has several limitations. First, the sample size was relatively small, which limits statistical power and the generalizability of the findings, particularly for the comparisons between polytrauma patients with and without nosocomial infections. In addition, no correction for multiple testing was applied, in accordance with methodological guidance for exploratory, hypothesis-generating studies, which recommends transparent reporting rather than formal adjustment procedures (30, 31). Second, although blood sampling was performed as consistently as possible within the constraints of acute trauma care, some variability in sampling times remained. Third, the control group consisted of patients undergoing elective instrumented posterior spinal fusion for adolescent idiopathic scoliosis rather than truly healthy controls. Nevertheless, this group was considered appropriate, previous research has not demonstrated associations between scoliosis and inflammatory outcomes (32). However, we acknowledge that acute stress related to the upcoming surgical procedure and preoperative medication (midazolam) may have influenced baseline immune measurements, introducing potential bias when comparing trauma patients to controls. Stress may induce low-grade inflammatory responses (33), whereas midazolam (administered in low doses to some patients) has been reported to exert mild anti-inflammatory effects (34). Finally, differences in age and gender between the study groups may have influenced immune measurements. Given the small sample size, formal adjustment for these potential confounders was not feasible, and their possible impact should therefore be considered when interpreting the findings. Despite these limitations, the findings appear internally consistent, with multiple independent outcomes reflecting the same overall patterns.

## 5 Conclusion

In summary, this study suggested that traumatic injury in paediatric patients rapidly elicits a robust immune response, particularly in cases of polytrauma, combined with early compensatory anti-inflammatory mechanisms. Moreover, children who later developed nosocomial infections appeared to show more pronounced and sustained immunosuppression. Given the overall small sample size and the exploratory nature of the analyses, particularly the subgroup analyses, these observations should be interpreted with caution and viewed as associative rather than causal. Immunosuppression was at least partially reversible upon *ex vivo* co-stimulation with IFN-γ. Future studies with larger cohorts are needed to validate these findings, to adjust for confounders, and to identify clinically meaningful threshold values distinguishing patients who will or will not develop nosocomial infections, which could help guide early recognition of children at risk and inform the selection of those who may benefit from targeted immune-stimulating interventions.

## Data Availability

The datasets presented in this article are not readily available because the data may be traceable to individual participants due to the small number of patients included, despite pseudonymization of the dataset. Requests to access the datasets should be directed to Stijn Nelen, stijn.nelen@radboudumc.nl.
