# Supplementary material for: Immune function in paediatric trauma patients: a prospective explorative cohort study
Source: Front Immunol. 2026 May 20;17:1814648. doi: 10.3389/fimmu.2026.1814648 (PMC13231501; doi:10.3389/fimmu.2026.1814648)
Supplement: Supplementary file 1 [file DataSheet1.docx]

Supplementary Material

# Supplementary Table 1 - Detailed information of laboratory analyses

| **Product** | **Supplier** | **Catalogue number** |
| --- | --- | --- |
| Sysmex haematology analyser | Sysmex Corporation, Kobe, Japan |  |
| Human HSP70/HSPA1A DuoSet ELISA kit | R&D systems, Minneapolis, MN, USA | DY1663-05 |
| Human S100A8/A9 Heterodimer DuoSet ELISA kit | R&D systems, Minneapolis, MN, USA | DY8226-05 |
| HMGB1 Express ELISA | TECAN, Männedorf, Switzerland | 30164033 |
| Luminex (MILLIPLEX® Human Cytokine/Chemokine/Growth Factor Panel A Magnetic Bead Panel) | Merck Millipore, Billerica, MA, USA | HCYTA-60K |
| *Escherichia coli* lipopolysaccharides | Sigma Aldrich, St. Louis, MO, USA | *E. coli,* serotype O55 |
| Recombinant Human IFN-γ (carrier-free) | Biolegend, San Diego, CA, USA | 570202 |
| RPMI 1640 medium (Dutch modified) | Thermo Fisher Scientific, Waltham, MA, USA | 22409031 |
| Gentamicin | Thermo Fisher Scientific, Waltham, MA, USA | 15750037 |
| Glutamax Supplement | Thermo Fisher Scientific, Waltham, MA, USA | 11574466 |
| Sodium pyruvate | Thermo Fisher Scientific, Waltham, MA, USA | 11530396 |
| Human IL-6 DuoSet ELISA | R&D Systems, Minneapolis, MN, USA | DY206 |
| Human IL-1 beta/IL-1F2 DuoSet ELISA | R&D Systems, Minneapolis, MN, USA | DY201 |
| Human TNF-alpha DuoSet ELISA | R&D Systems, Minneapolis, MN, USA | DY210 |
| Human IL-10 DuoSet ELISA | R&D Systems, Minneapolis, MN, USA | DY217B |
| BD Quantibrite™ Anti-Human HLA-DR PE/Monocyte PerCP-Cy™5.5 | Becton Dickinson, San Jose, CA, USA | 340827 |
| BD Quantibrite™ PE Phycoerythrin Fluorescence Quantitation Kit | Becton Dickinson, San Jose, CA, USA | 340495 |
| FACS Lysing Solution | Becton Dickinson, San Jose, CA, USA | 349202 |
| CytoFLEX flow cytometer | Beckman Coulter, Brea, CA, USA |  |

# Supplementary Table 1 - Comparison of immunosuppression in paediatric polytrauma patients with normal recovery (n = 6) versus those who developed nosocomial infections (n = 4). Data are presented as median (range).

|  |  |  | **Normal recovery**  **(n = 6)** | **Nosocomial infection**  **(n = 4)** | **p-value** |
| --- | --- | --- | --- | --- | --- |
| **Injury severity** | Injury Severity Score (ISS) | | 22 (17 – 26) | 27 (17 – 33) | 0.257 |
|  | Initial EMV score |  | 15 (4 – 15) | 5 (4 – 15) | 0.476 |
| **Immune cell counts and functionality** | Monocytes  (x 10^3^ cells/mL) | HEMS ER PID1 | 0.9 (0.4 – 1.2) 0.8 (0.6 – 1.4) 0.7 (0.5 – 1.5) | 1.1 (0.7 – 3.1) 0.9 (0.6 – 1.8) 2.1 (0.8 – 3.6) | 0.352 0.610 0.038 |
|  | mHLA-DR  (antibodies/cell) | HEMS ER PID1 | 38145 (33173 – 48998) 26820 (18208 – 30599) 15950 (14000 – 23644) | 26103 (20948 – 30992) 14970 (11899 – 22918) 6297 (4280 – 13005) | 0.010 0.038 0.010 |
| ***Ex vivo* cytokine production (LPS)** | IL-1β (pg/mL) | HEMS ER PID1 | 1817 (613 – 3017) 572 (366 – 867) 1027 (504 – 2176) | 1292 (1158 – 1514) 217 (117 – 706) 170 (61 – 364) | 0.714 0.381 0.024 |
|  | TNF (pg/mL) | HEMS ER PID1 | 809 (173 – 1882) 293 (204 – 529) 539 (460 – 914) | 364 (322 – 519) 89 (68 – 176) 233 (210 – 252) | 0.262 0.024 0.024 |
|  | IL-6 (pg/mL) | HEMS ER PID1 | 14509 (3826 – 20773) 8198 (4387 – 13565) 10589 (7308 – 14361) | 14027 (10220 – 28415) 3958 (3776 – 5321) 6305 (5672 – 11901) | 0.714 0.048 0.167 |
|  | IL-10 (pg/mL) | HEMS ER PID1 | 210 (133 – 364) 107 (78 – 170) 218 (95 – 520) | 125 (74 – 171) 54 (37 – 77) 110 (97 – 301) | 0.095 0.024 0.548 |
